# Active C4 Electrodes for Local Field Potential Recording Applications

**DOI:** 10.3390/s16020198

**Published:** 2016-02-04

**Authors:** Lu Wang, David Freedman, Mesut Sahin, M. Selim Ünlü, Ronald Knepper

**Affiliations:** 1 Department of Electrical and Computer Engineering, Boston University, 8 Saint Mary’s St, Boston 02215, MA, USA; dsf@bu.edu (D.F.); selim@bu.edu (M.S.Ü.); rknepper@bu.edu (R.K.); 2 Department of Biomedical Engineering, New Jersey Institute of Technology, 323 Martin Luther King, Jr. Boulevard, University Heights Newark, Newark 07102, NJ, USA; sahin@njit.edu; 3 Department of Biomedical Engineering, Boston University, 44 Cummington St, Boston 02215, MA, USA

**Keywords:** 3D electrodes, C4, CMOS, extracellular, *in vitro*, *in vivo*, MEA, neural sensors

## Abstract

Extracellular neural recording, with multi-electrode arrays (MEAs), is a powerful method used to study neural function at the network level. However, in a high density array, it can be costly and time consuming to integrate the active circuit with the expensive electrodes. In this paper, we present a 4 mm × 4 mm neural recording integrated circuit (IC) chip, utilizing IBM C4 bumps as recording electrodes, which enable a seamless active chip and electrode integration. The IC chip was designed and fabricated in a 0.13 μm BiCMOS process for both *in vitro* and *in vivo* applications. It has an input-referred noise of 4.6 μV${}_{\mathrm{rms}}$ for the bandwidth of 10 Hz to 10 kHz and a power dissipation of 11.25 mW at 2.5 V, or 43.9 μW per input channel. This prototype is scalable for implementing larger number and higher density electrode arrays. To validate the functionality of the chip, electrical testing results and acute *in vivo* recordings from a rat barrel cortex are presented.

## 1. Introduction

Extracellular recordings of neural activity with an array of electrodes placed on the brain surface or juxtaposed to an extracted brain slice have been shown to be very useful for studying neural function at the network level [1,2]. To achieve high spatial resolution, high density multi-electrode arrays (MEAs) are often used for extracellular recordings [3,4,5]. These MEAs can record the low frequency field potentials generated by local neurons, and sometimes high frequency neural spikes if they are sufficiently close to individual cells, even though the extracellularly recorded neural signals are on the order of tens of microvolts. Both local field potentials (LFP) at the lower end of the frequency spectrum and the multi-unit activity in the kilohertz region contain functional information [6,7]. There is an increasing demand for larger count electrodes with increased spatial density in order to collect the maximum amount of information from the neural tissue of interest. Large amplification and low input-referred noise are also required due to the low amplitudes of extracellular potentials. Additionally, power density must be kept below 0.8 mW/mm${}^{2}$ to prevent damage to the surrounding neural cells [8]; this requirement demands minimal power dissipation from the system [9].

High density active MEAs with small chip areas have been developed to achieve high spatial resolution [10,11,12]. Usually a large amount of post processing is required to fabricate the 2D electrodes on top of a standard CMOS chip. Passive MEAs with 3D electrodes, such as the Utah electrode array (UEA) [8,13,14,15,16,17], and flexible or rigid multi-shank electrode arrays [18,19,20,21], have been integrated with CMOS integrated circuits (ICs) to create active MEAs. A capacitive feedback pre-amplifier topology [22] has been widely adopted in the neural recording system design [15,23,24,25]. The voltage gain of this pre-amplifier is determined by the ratio of its input coupling capacitor and the feedback capacitor. However, the use of this approach also limits its scalability, since the capacitance per unit area does not increase linearly as CMOS technology scales down. Critically, commercially available UEAs have a maximum of 100 electrodes with a minimum pitch of 400 μm, which further limits the scalability of such active MEAs.

**Figure 1:**
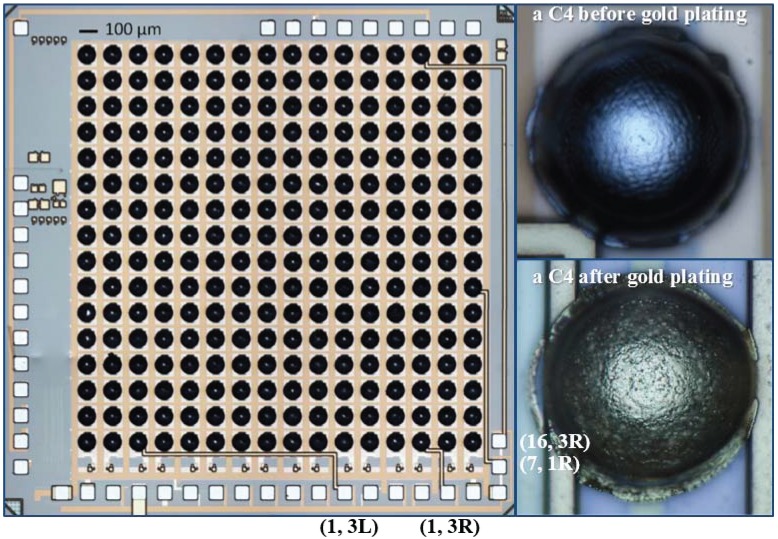
Micrograph of the fabricated neural recording integrated circuit (IC). The input channels with direct connections to the package are all labeled near the chip’s corresponding pads. The C4 image is compiled from a number of different focus points on the microscope; *i.e.*, to see the whole C4, this and the previous C4 images were assembled from multiple Z focus-positions

In this paper, we present the characterization and preliminary animal testing results of a scalable, CMOS compatible, neuropotential recording IC with C4 (controlled collapsed chip connect) solder bumps as 3D electrodes, as shown in Figure 1. The recording system has AC-coupled single-ended input channels with PMOS transistors biased in the accumulation region as pseudo-resistors to achieve sub-Hertz low-frequency bandwidth, and uses the cascode transistor of the input stage as a switch, which enables input channel multiplexing for data serialization and tail current reuse for power reduction. Current conveyor circuitry is incorporated into the design to achieve high voltage gain with improved stability. This is the first time C4s have been demonstrated as electrodes for *in vivo* recording.

## 2. Chip Design

**Figure 2:**
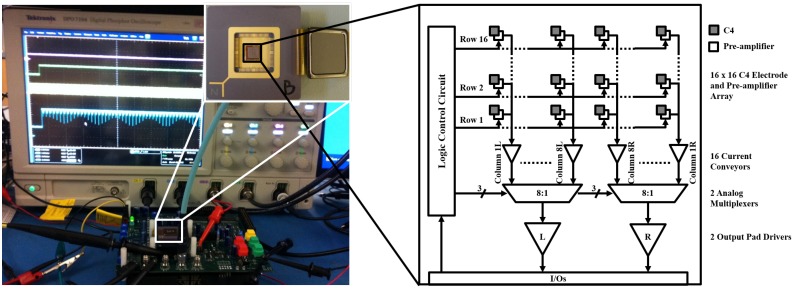
Neuropotential acquisition system and block diagram of the neuropotential acquisition IC chip architecture. For the prototype, the chip was designed with two analog outputs, and the digitization and signal processing are performed on the board level to reduce the design complexity.

The architecture of the neuropotential recording IC chip is shown in Figure 2. The active MEA has 256 on-chip C4 electrodes for extracellular recording, occupying an area of 3.2 mm × 3.2 mm (active area), or 25 electrodes per mm${}^{2}$. Each C4 electrode is capacitively-coupled to a low-noise pre-amplifier to form an input channel. The AC coupling can prevent the system from becoming saturated by the half-cell potential of the electrode contacts. Sixteen rows are selected sequentially by an on-chip counter, and two analog multiplexers are used to select one of eight columns on both the left and right sides. At any given time, only one column on each side of the chip (left and right) is connected to an output channel, and thus only two output channels are needed for the 256 input channels. In the following sections, an input channel row x, column y on the left side will be referred as input (x, yL). Testing was performed on a custom-designed printed circuit board (PCB).

### 2.1. C4 Electrodes

C4s are standard IBM solder balls used in flip-chip bonding, with different sizes and pitches available. The smallest size available at the time of this chip fabrication is “3 on 6” (mil), which has a diameter of 75 μm and a pitch of 150 μm [26]. This chip is designed using a 130 nm technology that comes with the C4 option of “4 on 8”, so each C4 electrode occupies a chip area of 0.04 mm${}^{2}$. This area is much larger compared to individual pre-amplifiers and even the metal-insulator-metal (MIM) capacitors, which offer about 1fF per μm${}^{2}$ in this technology, so the C4 or any other electrode is often the major limiting factor of the MEA density. For example, with “2 on 4” C4s [26], the same chip area will be able to accommodate 1024 electrodes. Continued development is increasing pitch densities, *i.e.*, the most advanced fine pitch Cu pillar bumps have a staggered pitch of 30/60 μm [27].

An advantage of using C4s as on chip electrodes is that they are compatible with the standard CMOS process, and minimum post processing is required, which leads to low cost and high yield. Also, for single unit recording, it is possible to integrate penetrating electrodes with the presented IC by flip-chip bonding [15].

**Figure 3:**
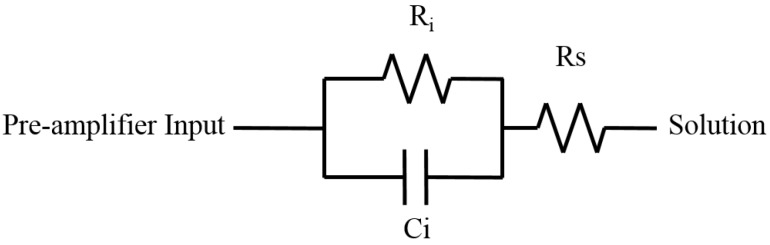
Equivalent circuit model for C4 electrode.

The equivalent circuit model used for a C4 electrode in this work is shown in Figure 3 and was adopted from [28,29,30]. R${}_{i}$ and C${}_{i}$ represent the electrode interface resistance and capacitance, respectively. R${}_{s}$ models the spreading resistance. R${}_{i}$ models the charge transfer resistance and has been theoretically determined to be given by the low-field approximation to the Butler-Volmer equation [28]. C${}_{i}$ is derived from the determination of the constant phase angle impedance Z${}_{\mathrm{CPA}}$ [28]. However, in this work, both R${}_{i}$ and C${}_{i}$ were determined from measurements of C4 impedance, which will be reported later in Section 4.3.

The spreading resistance R${}_{s}$ will depend to some extent upon the position of the C4 on the 16 × 16 array. For this work we use a calculated value of 3.18 KΩ, determined from the expression $R_{s}=\rho_{s}/2\pi r_{ei}$, where the resistivity of the solution $\rho_{s}=100\;\Omega -\mathrm{cm}$, and the radius r${}_{\mathrm{ei}}$ of the C4 electrode is 50 μm.

### 2.2. Low-Noise Preamplifier

**Figure 4:**
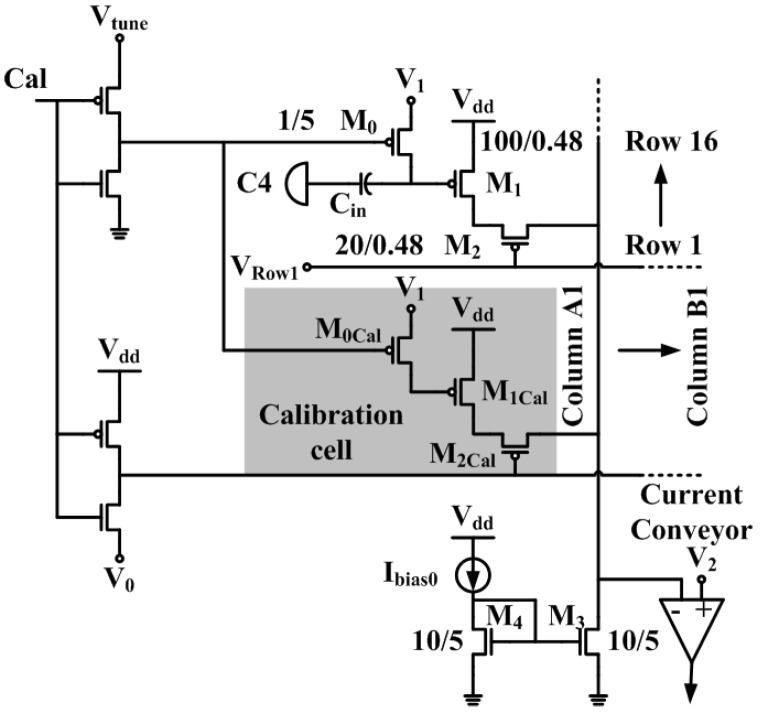
Circuit schematic of the pre-amplifier array. The calibration cell has the same size as a regular pre-amplifier, only without being connected to a C4 input electrode. There is one calibration cell on each column. The substrates of NMOS and PMOS devices are connected to the ground and the 2.5 V power supply, respectively.

The circuit schematic of the pre-amplifier array is shown in Figure 4. Each pre-amplifier from the array is a single-ended input PMOS cascode amplifier. The input of each pre-amplifier is AC coupled to the C4 recording electrode through a 10 pF MIM capacitor C${}_{\mathrm{in}}$. The input of the pre-amplifier is also DC coupled to a fixed voltage V${}_{1}$ (*i.e.*, 1.8 V) through a high-impedance pseudo-resistor M${}_{0}$ to provide biasing. The PMOS pseudo-resistor can achieve a GΩ-TΩ resistance value R${}_{0}$ with transistor M${}_{0}$’s gate biased in the sub-threshold region [18,22], or even in the accumulation region [31]. The low frequency corner f${}_{L}$ of the pre-amplifier is determined by the dominant capacitor C${}_{\mathrm{in}}$ and the pseudo-resistor R${}_{0}$. Its equation is given by

(1) $$f_{L}=\frac{1}{2\pi C_{in}R_{0}}$$

The function of the calibration cell will be discussed in Section 2.4.

To achieve the desired f${}_{L}$ value, a larger R${}_{0}$ allows the use of a smaller input coupling capacitor. Theoretically, to the first-order approximation,

with a TΩ resistor, a 160 fF capacitor is enough to achieve a 1 Hz low frequency corner. A single-ended input topology with a high resistance pseudo-resistor doesn’t rely on large value capacitors, and thus allows this approach to take advantage of further scaling.

The input-referred thermal noise voltage of the pre-amplifier is:

(2) $$\bar{v_{n}^{2}}=\frac{8kT}{3g_{m1}}\cdot \left(1+2\cdot \frac{g_{m3}}{g_{m1}}\right)\cdot \Delta f$$

Therefore, to reduce the thermal noise, a large g${}_{m1}$ and a small g${}_{m3}$ is desired. In terms of transistor size, a larger gate width over a length ratio of M${}_{1}$ (100 μm / 0.48 μm) was used, compared to M${}_{3}$ (10 μm/5 μm). There is a trade-off between minimizing the thermal noise and the 1/f noise, since the latter requires a small g${}_{m1}$ value.

The input-referred root mean square (rms) noise voltage is calculated using the noise frequency curve by

(3) $$\bar{v_{rms}}=\sqrt{\int_{10Hz}^{10kHz}\frac{\bar{v_{n}^{2}}}{\Delta f}df}$$

which gives 3.99 μV${}_{\mathrm{rms}}$.

### 2.3. Current Conveyor

**Figure 5:**
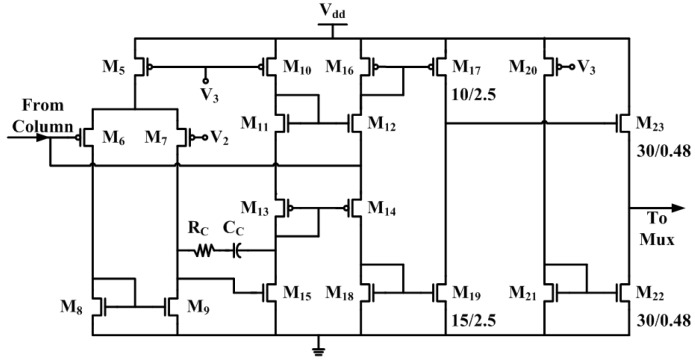
Circuit Schematic of the current conveyor amplifier.

The output of the pre-amplifier is connected to the current conveyor. As shown in Figure 5, the current conveyor is a current amplifier followed by a transimpedance structure (M${}_{16}$–M${}_{19}$) and an NMOS source follower. With the inverting input of the differential stage connected to a feedback loop, the DC bias voltage follows the non-inverting input V${}_{2}$ that is supplied from an on-chip voltage reference. This is the primary reason a current conveyor is used here because the voltage of the inverting input is the drain voltage of transistor M${}_{3}$ of the pre-amplifier array. As shown in Figure 4, all 16 pre-amplifiers in a column share the same transistor M${}_{3}$, so any change in the drain voltage of M${}_{3}$ will affect the performance of the whole column. The current conveyor stage is essentially a transimpedance amplifier, and its gain is heavily dependent on the output impedance of transistor M${}_{17}$ and M${}_{19}$, which convert the current signal back to the voltage domain, so a transistor length of 2.5 μm is used for both M${}_{17}$ and M${}_{19}$. The total voltage gain A${}_{v}$, on the first order, is

(4) $$A_{v}=g_{m1}\times (r_{o17}\left|\right|r_{o19})$$

where g${}_{m1}$ is the transconductance of transistor M${}_{1}$ in Figure 4. The total gain of the first two stages is simply the input transistor transconductance times the output impedance, because the current conveyor mirrors the signal current with a ratio of 1:1.

The signals generated by the logic control circuit are non-overlapping, which means there will be a short time when all the pre-amplifiers in a column are shut down. The existence of the current conveyor will provide a DC path to V${}_{\mathrm{dd}}$ for the drain of M${}_{3}$ during this time. This path will prevent the drain of M${}_{3}$ from being pulled down to ground. Otherwise, switching between rows would cause severe voltage spikes on the output waveform and most importantly would slow down the frame readout speed of the neural recording chip.

To ensure stability, a dominant-pole compensation capacitor C${}_{c}$ is used to control the dominant first pole and to split it from the high frequency poles [32] so that the open-loop gain of the amplifier’s transfer function will be brought down to unity before the frequency reaches the high frequency poles. Also, the resistor R${}_{c}$ is included to introduce a left-half-plane zero above the unity gain frequency in order to relax the high frequency poles’ effect on the phase. A simulated phase margin of 86.65${}^{\circ }$ was realized with this RC compensation technique.

### 2.4. Time-Division Multiplexing and Chip Operation Modes

**Figure 6:**
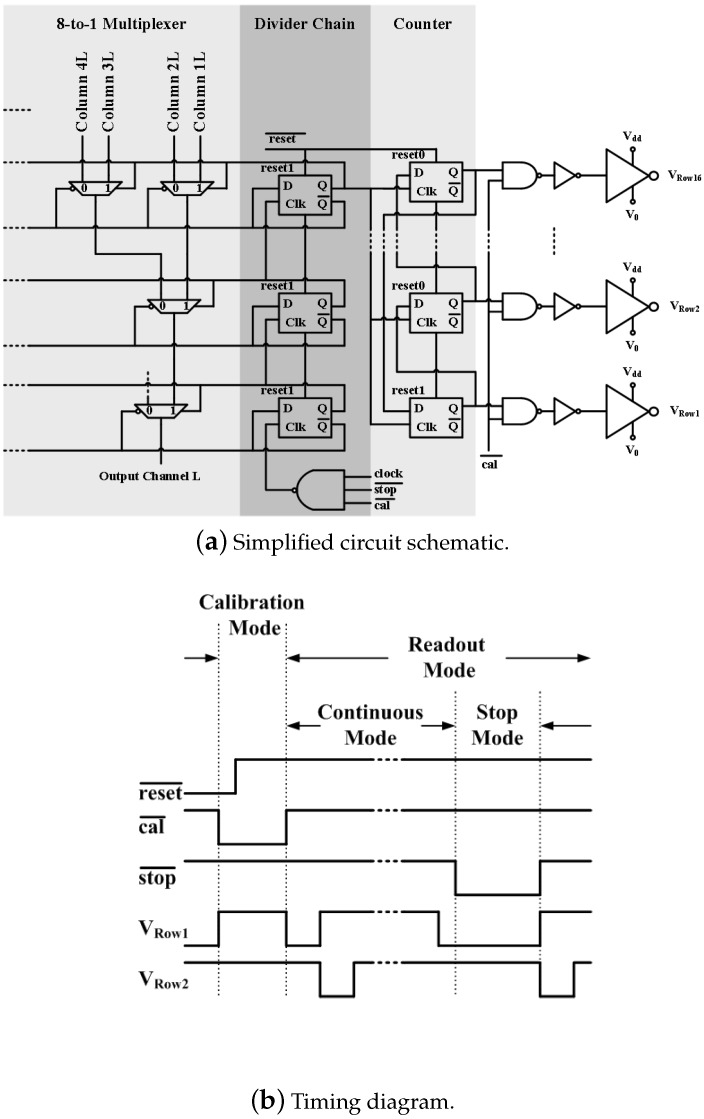
The logic control circuit. (**a**) The schematic shows only half of the 8-to-1 multiplexer. The $\bar{\mathrm{reset}}$ signal is routed through some of the D-latches simply to reduce clutter in the figure; (**b**) The timing diagram is an illustration of the chip’s operation modes; the clock and all the control signals $\bar{\mathrm{reset}}$, $\bar{\mathrm{cal}}$, and $\bar{\mathrm{stop}}$ are provided off-chip.

For high density MEAs, time-division multiplexing (TDM) [18] and sample-and-hold (SH) scheme [15] are often used to reduce the total number of output channels. Additionally, further digital compressive modulation schemes are often required for wireless systems because of their restrained bandwidth [33,34,35]. In this paper, for simplicity, TDM is utilized, so 256 signals recorded by the input amplifiers will be sequentially multiplexed onto 2 output channels to minimize the power consumption, chip area, and the number of chip I/Os. As shown in Figure 6a, a synchronous logic control circuit is used to carry out the TDM scheme. Every time when the chip is powered on, the low enabled $\bar{\mathrm{reset}}$ signal is asserted to perform a power-on reset (POR). During POR, the $\bar{\mathrm{cal}}$ and $\bar{\mathrm{stop}}$ pins are kept high, the output of the “1” hot counter will be reset to its initial value 1, so V${}_{\mathrm{Row}1}$ is set to V${}_{0}$ (around 0.9 V) (On-chip current mirror bias circuits establish voltages V${}_{0}$–V${}_{3}$ and V${}_{\mathrm{cal}}$ through off-chip current sources that have nominal values of 100 μA. The tuning range of the current source is 0 to 200 μA. V${}_{\mathrm{tune}}$ is normally set at 2.5 V.), whereas V${}_{\mathrm{Row}2}$ to V${}_{\mathrm{Row}16}$ all equal V${}_{\mathrm{dd}}$. V${}_{\mathrm{Row}1}$ to V${}_{\mathrm{Row}16}$ are used to control the gate of the cascode transistors M${}_{2}$, as shown in Figure 4, and thus during POR (or reset mode in general), only row 1 will be selected. Similarly, during POR, the output of the divider chain is reset to 111, and the 8-to-1 multiplexer connects column 1 to the output channel, so one column is selected from each side of the chip.

After POR, the $\bar{\mathrm{reset}}$ pin is deasserted, the output of the divider-chain will count from 111 to 000 every 8 clock cycles, whereas the output of the counter repeats from 1 to 16 every 128 clock cycles. Thus, a consecutive input channel is selected every clock cycle, from input (1, 1) to (16, 8). Since at any given time only one of the M${}_{2}$ transistors in a column is selected, all the pre-amplifiers in a column can share the same tail-current. As a result, the power consumption of the pre-amplifier array is reduced by a factor of 16. This is the continuous mode. The clock is designed to operate at 2.56 MHz in continuous mode, so each input channel will be selected every 128 clock cycles, for a 20 kHz sampling rate per channel (The design scan rate of 2.56 MHz has not been achieved in testing due to the impact of process variation. This will be discussed in Section 4).

The chip can also operate in two additional modes: stop mode and calibration mode. Pulling down the $\bar{\mathrm{stop}}$ pin will enable the stop mode, whereby the clock will be superseded, interrupting the TDM. Thus, stop mode allows observation in real time of any channel from each half of the chip. Continuous mode is resumed once the $\bar{\mathrm{stop}}$ signal is disabled. After every reset, a calibration often needs to be performed. As mentioned previously, the pseudo-resistor enables the recording of LFPs with small input capacitors. However, this high resistance value results in a relatively large RC time constant, which slows down the process of charging and discharging the gate of the M${}_{1}$ transistor. In order to accelerate this process, the $\bar{\mathrm{cal}}$ signal is used to turn on M${}_{0}$ to convert the pseudo-resistor into a low resistance state to acquire a desirable RC time constant that can calibrate the gate voltage of M${}_{1}$ to V${}_{1}$ efficiently. During calibration, the gate of M${}_{2\mathrm{Cal}}$ (as shown in Figure 4) will be pulled down to V${}_{0}$ to activate the calibration pre-amplifiers, while all the regular pre-amplifiers are disabled. The purpose of the calibration pre-amplifiers is to avoid the drain voltage of M${}_{3}$ being pulled down to ground during calibration. By keeping the drain voltage of M${}_{3}$ stable, it is possible to increase the switching speed between operation modes. The timing diagram of the operation modes is plotted in Figure 6b.

## 3. Fabrication and Packaging

The neural recording chip was designed and fabricated in IBM 0.13 μm BiCMOS 8HP technology (No bipolar devices were used in the chip in order to keep the design CMOS compatible). It has a total area of 4 mm × 4 mm and a recording area (active area) of 3.2 mm × 3.2 mm. A micrograph of the chip is shown in Figure 1. The C4 electrodes are standard “4 on 8” (mil), 100 μm in diameter with a 200 μm pitch. Their material composition is 97% Pb (lead) and 3% Sn (tin) (Lead free version of C4s are also available with 97.7% Sn and 2.3% Ag (silver). Scheduling issues necessitated using Pb-based C4s on this chip). To improve the C4 electrode biocompatibility, gold was plated onto the C4s using Bright Electroless Gold solution (Transene Company, Inc.) [36,37]. The photographs of C4s before and after Au electrolessplating are shown in Figure 1.

**Figure 7:**
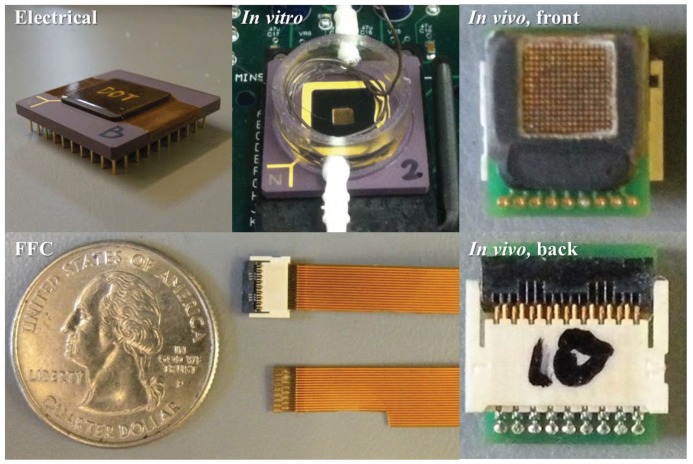
Chip packaging: A standard 10 × 10 ceramic PGA (**Top left**), an open-top 10 × 10 ceramic PGA (**Top middle**), front side of the custom printed circuit board (PCB) package (**Top right**), back side of the custom PCB package (**Bottom right**), and flat flexible cables (FFC) (**Bottom middle**).

In order to accommodate the different requirements of electrical, *in vitro*, and *in vivo* testing, the fabricated chips were wire bonded to three different packages (Quik-Pak) [38], as shown in Figure 7. For electrical testing, a 10 × 10 ceramic PGA package was used to provide direct access to input channels (1, 3L), (1, 3R), (7, 1R), and (16, 3R) that were pre-wired to the chip pads with the top level metal, as shown in Figure 1. For *in vitro* applications, the neural recording chip was packaged in an open-top PGA package with bonding wires encapsulated for mechanical and electrical protection. The finished package has all the C4 electrodes exposed in order to interface with the brain/tissue slices, and a plastic fluidic chamber was built on top of the PGA package. An important function of the *in vitro* testing package is to provide a way to apply signals to all 256 input channels.

A 6 mm × 7 mm custom PCB package was also built to carry out acute *in vivo* experiments in living rats. The diameter of a rat brain cross-section is not much bigger than 1 cm, so the major requirement of the *in vivo* package is that it has to be able to fit on top of the rat brain. Meanwhile, it has to be connected to the recording side with a minimum number of wire connections without completely constraining the rat under test. In order to do so, the chip was wire bonded to the top side of the PCB with the bond wires encapsulated. The height of the epoxy was milled down to 100 μm after the encapsulation for better contact between the electrodes and rat brain. A 0.3 mm pitch, 17 pin flat flexible cable (FFC) was chosen as the interconnection between the *in vivo* package and a recording PCB. The width of the FFC was 5.1 mm, which is slightly smaller than the width of the PCB package, and is thin and flexible, all of which makes the FFC suitable for acute *in vivo* testing.

## 4. Chip Characterization

### 4.1. Electrical Testing

#### 4.1.1. System Voltage Gain

**Figure 8:**
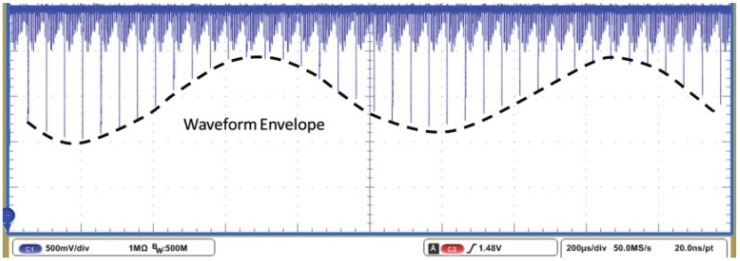
Measured output waveform from the recording chip in continuous mode with a sampling rate of 20 kHz. The envelope of a single channel that had a 1 kHz sinusoidal input is highlighted with the dashed line.

The electrical testing package was used to characterize the voltage gain of a single input channel. Figure 8 shows the measurement when the IC is operating in continuous mode at the maximum frame rate of 20 kHz. 128 inputs (per side) were scanned with only one input channel connected to the function generator. A sinusoidal signal with a peak-to-peak amplitude of 1 mV and a frequency of 1 kHz was applied to input channel (1, 3L) through an subminiature version A (SMA) connector. All the other input channels were left floating. The sine wave at the output can be seen in the highlighted envelope of the selected cell with the provided input. Calculation using the amplitude of the waveform envelope gives a voltage gain of 58.1 dB.

All 128 input channels on the left side of the chip were measured in stop mode using the *in vitro* testing package. The output DC voltage level of each input channel was manually adjusted to 1.5 V. The mean and standard deviation of the voltage gain of the 128 inputs on the left side of the chip were 58.7 dB and 0.37 dB, respectively.

#### 4.1.2. Process Variation

**Figure 9:**
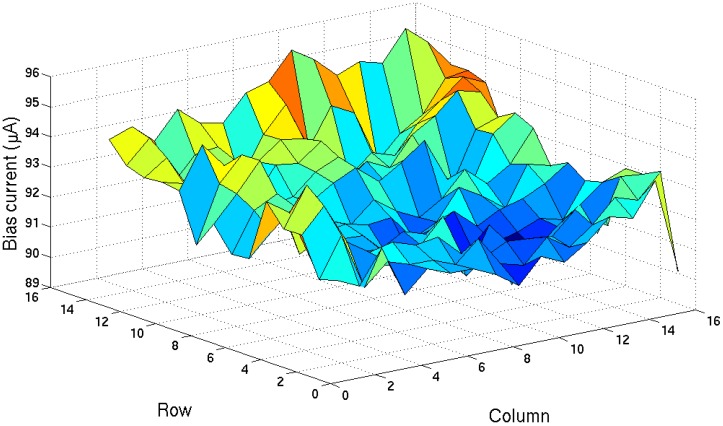
Bias current values to set the output DC level of each individual input channel to 1.5 V across the entire chip.

Semiconductor process variation is the deviation of the manufactured device or interconnect parameters from their designed or expected nominal values. It can cause mismatch in current mirrors, different input recording channels, and threshold voltage variations. As a result, the chip output DC level will vary from channel to channel. In order for the neuropotential recording chip to work properly, the output DC level needs to be corrected for each individual input channel. This can be done by adjusting the off-chip current sources as shown in Figure 9. However, to operate the chip in continuous mode, the variation correction process needs to be integrated into the recording PCB so that during every clock cycle a proper bias current will be applied to the chip to automatically calibrate the DC voltage value of the output channel.

**Figure 10:**
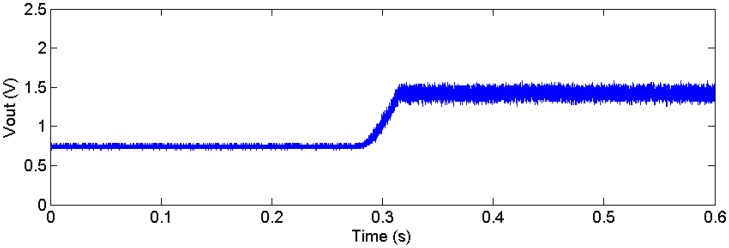
A demonstration of automatic correction for a single channel in stop mode.

Currently, we are using a microcontroller to provide a clock signal to the MEA chip, as well as to control the bias current value. Ideally, this should be performed on-chip to prevent the I/O interface latency, which determines how fast the bias current can be changed and limits the clock’s maximum frequency. Figure 10 shows the measured results from preliminary work on the process variation correction PCB system. The output voltage of the chip is digitized and used to control the chip’s bias current. At t = 0, since V${}_{\mathrm{out}}$ is smaller than 1.5 V, the feedback increases the bias current by 0.1 μA per step, which in turn increases the output DC voltage (V${}_{\mathrm{out}}$ ramp at 0.3 s in Figure 10). At 0.32 s, the output DC voltage reaches 1.5 V, and the feedback system locks and holds the bias current constant. This calibration procedure is done once at power-on, and the corresponding I${}_{\mathrm{bias}0}$ values for each input are recorded in the microcontroller for use in the continuous mode testing operation. However, this off-chip method requires an excessive amount of time to adjust the output offset for each individual input during the continuous mode, so the continuous mode feature cannot be operated at the expected 10 kHz rate.

#### 4.1.3. Frequency Response and Noise Performance

**Figure 11:**
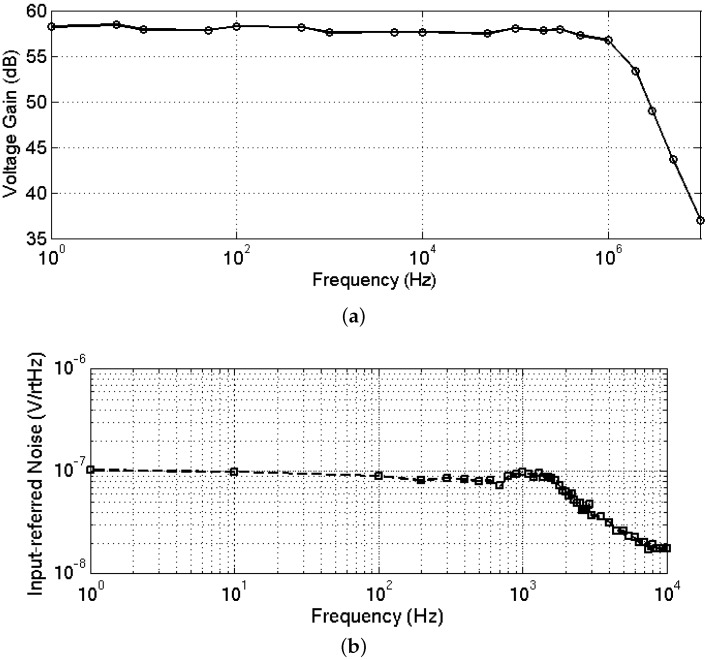
Measured system (**a**) frequency response; and (**b**) input-referred noise.

The frequency response and input-referred noise voltage spectral density of the neural recording IC were measured with both electrical and *in vitro* testing packages using an oscilloscope and a spectrum analyzer, respectively. The stimulus was provided to the IC through an SMA connection. As shown in Figure 11, the system frequency response has a high frequency cutoff of 1.4 MHz. The low frequency corner is below 0.05 Hz, which is beyond the range of our spectrum analyzer, indicating that the pseudo-resistor has a resistance value greater than 0.32 TΩ. The input-referred noise spectrum was measured in stop mode, and the integrated input-referred noise voltage is 4.6 μV${}_{rms}$ for a 10 Hz to 10 kHz bandwidth. As a result of the process variation issue, this measurement could not be done in the continuous mode at a 20 kHz sampling rate, due to the length of time required to center the output for each input channel. Therefore, the measurements reported here do not include any potential contribution from noise folding, clock noise, and crosstalk.

### 4.2. Electrical Testing With the In Vitro Package

**Figure 12:**
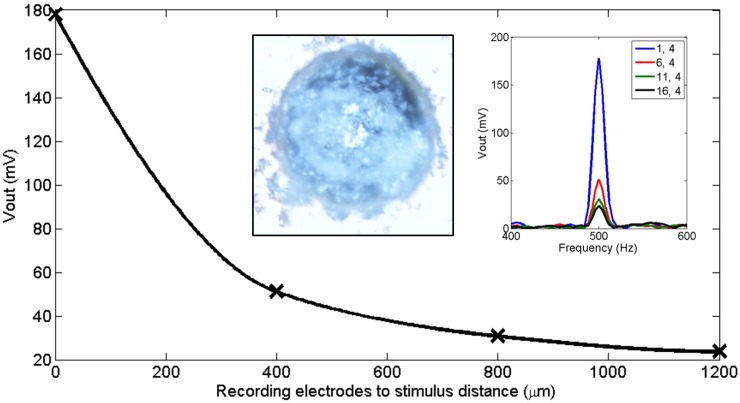
Measured output voltage amplitude with respect to the distance between the recording electrodes and the stimulation electrode. The inset on the right shows the output frequency spectrum, and the inset on the left shows a white substance built up on the surface of a gold plated C4 after electrical testing with the *in vitro* testing package.

To record neural signals at the network level, which has significant importance in interpreting brain activities [1], the recording IC has to be sensitive to the signal source locations. To demonstrate the IC’s location mapping ability, a 500 Hz sinusoidal signal was applied to a drop of phosphate buffered saline (PBS) solution by a tungsten electrode near the bottom of the chip, with the PBS solution grounded by a silver electrode coated with silver chloride (Ag/AgCl) around the chamber edge. As shown in Figure 12, the amplitudes of the recorded signals decrease as the distance between the C4 recording electrodes and tungsten electrode increases.

### 4.3. C4 Impedance Measurements

AC impedance measurements were made between the two C4 inputs (1, 3L) and (1, 3R), using the standard 10 × 10 ceramic PGA chip package along with the miniature *in vivo* PCB and flexible cable interface, as shown in Figure 7. The measurements were made with the *in vitro* package by pipetting saline solution within the fluidic chamber on top the C4s to provide conductivity between the two given C4 electrodes. C4 inputs (1, 3L) and (1, 3R) are brought out to external pads and wired via bond wires to I/O pins so that they can be separately accessed. The measurements were made using a low-frequency signal generator to provide a signal input to one C4 and a transimpedance op-amp connected to the other C4 in order to measure the AC current.

Measurements were made on 7–8 multiple-input neuron sensor (MINS) chips over the frequency range of 1 kHz to 10 kHz. Assuming symmetry between both C4s, values of R${}_{i}$ = 725 kΩ and C${}_{i}$ = 620 pF were obtained for the model of a single C4, as shown in Figure 3. The much smaller value of R${}_{s}$ = 3.18 kΩ is seen to have little impact on the overall impedance, given the large value of the interface resistance, R${}_{i}$. It should be noted that the particular IBM 8HP fabrication run, from which the chips used in this work were obtained, contained leaded C4s. We believe the salt buildup was from incomplete gold-plating and a non-revisable reaction of the solution ions with the leaded C4s. Any future work with a MINS-derivative IC should be done with non-leaded C4s, *i.e.*, Ag/Tin C4s.

## 5. Acute Rat Cortical Recording

### 5.1. Surgical Procedure

**Figure 13:**
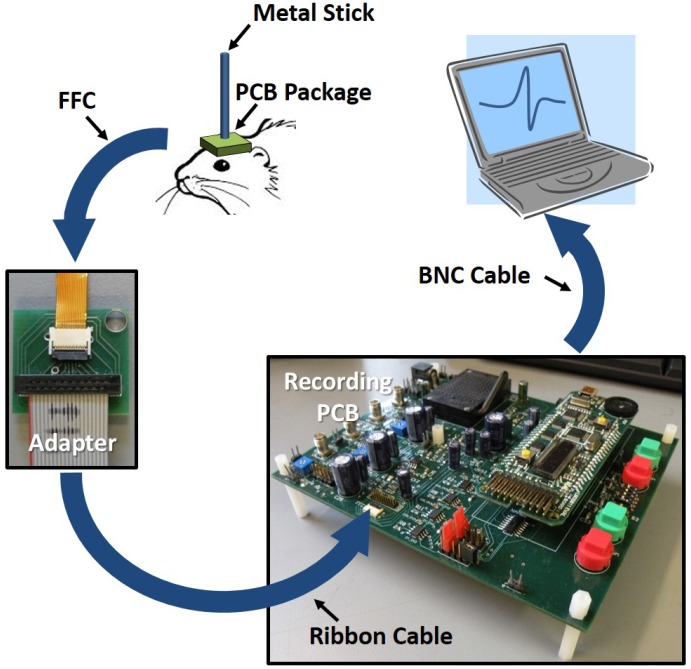
Illustration of the rat cortical recording setup.

The fabricated array was tested in a Sprague-Dawley rat (∼400 g) under ketamine and xylazine (80 mg/kg and 12 mg/kg, respectively, IP) anesthesia. All procedures were approved and performed in accordance with the guidelines of the Institutional Animal Care and Use Committee, Rutgers University, Newark, NJ. The fur over the skull was shaved, and the animal was placed in a stereotaxic frame. The body temperature was kept at around 36 ${}^{\circ }$C with the help of a heating pad under the animal. The blood oxygenation was continuously observed with a pulse oximeter from the hind paw while the animal breathed spontaneously. The skull over the barrel cortex was removed in a 4 mm × 4 mm rectangular area on the right side. The dura was cut and reflected over to expose the cortical surface. Normal saline was applied to keep the cortex moist. The array was attached to a stainless steel rod in the middle of its top surface using medical epoxy (Figure 13). The array was attached to a micromanipulator by the metal rod for easy positioning and mechanical stability. The reference electrode was a platinum wire soldered to the array ground and dipped into the saline pool around the array. The array was slowly lowered until all contacts (C4 bumps) were touching the barrel cortex.

### 5.2. Recording Procedures

**Figure 14:**
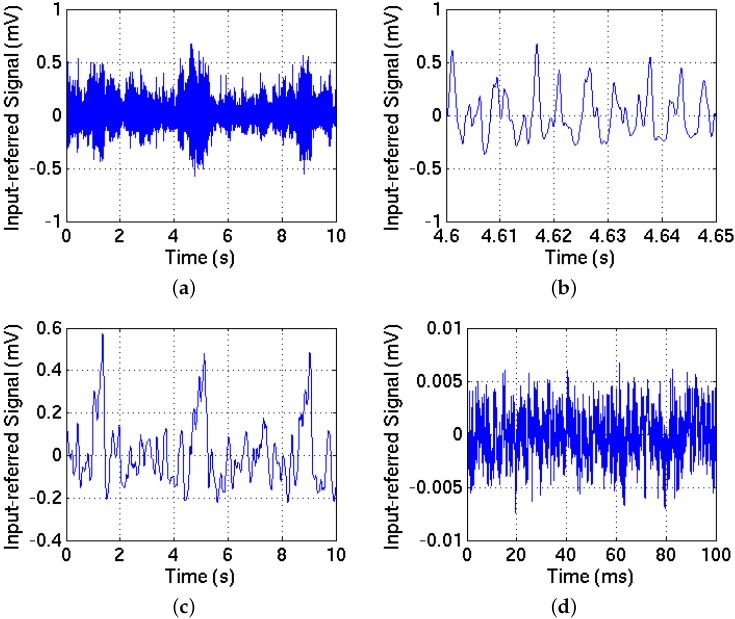
An episode of neural signals recorded in the rat barrel cortex compared to the signal recorded with grounded input. (**a**,**b**): A 300–3000 Hz band-pass filtered version of the signals shows the multi-unit activity—(**b**) is the zoomed view of (**a**); (**c**): A 1–30 Hz band-pass filter was applied to the same data to extract the LFPs; (**d**): The control signal was recorded with the input grounded. A 3000 Hz low-pass filter was applied (The DC voltage was subtracted for clarity of presentation).

The multiplexed outputs of the recording array were connected to a National Instruments Data Acquisition Board (PCI-6071), and the neural signals were acquired at 20 kHz in 10 s episodes into a desktop computer. Another Matlab code initialized the board and selected one of the 256 contacts for neural recording. Multi-unit activity, as well as LFPs, were recorded as shown in Figure 14a,b, respectively. All the data were collected within an hour after performing the surgical procedure on the rat. Visible C4 imprints were observed on the surface of the rat brain after recording. The control signal (Figure 14d) was recorded with the input grounded through a PCB connector. The control signal is two orders of magnitude smaller than the measured LFP signals.

## 6. Discussion and Conclusions

An active C4 electrode array for LFP recording has been demonstrated with a mean voltage gain of 58.7 dB and a standard deviation of 0.37 dB after output DC level correction. It has a scalable topology by minimizing the use of on-chip capacitors, while utilizing high-value PMOS pseudo-resistors to extend the low frequency corner. The on-chip C4 3D electrodes are compatible with standard CMOS technology and require minimum post processing. To record low-frequency and low-amplitude neural signals, a sub-Hertz low-frequency corner and 4.6 μV${}_{\mathrm{rms}}$ input-referred noise voltage have been achieved. The power consumption of the chip was minimized to 11.25 mW to prevent neural damage caused by heat. Additionally, the recording chip has been validated with *in vivo* animal recording.

In this paper, we demonstrated the use of C4 solder bumps as neural recording electrodes. The MEA was originally designed for both LFP and single unit recording applications. However, our *in vivo* measurements with a live rat were predominately those of LFP signals, apparently due to the distance between the spiking neurons and the C4 electrodes on the surface. For single unit recording use, the chip can be designed with smaller size C4s, when available, or it could be integrated with other penetrating electrodes. Due to the use of a phosphate buffered saline solution during testing , a white substance continually built up on the surface of the gold plated C4s (Figure 12), which significantly increased the resistance values of the C4s when used for a period of time. Eventually, the electrodes became unsuitable for signal recording. So, it is highly recommended to use lead-free C4s, coated with gold, as recording electrodes [36].

Additionally, at this stage, the MINS IC is more suitable for LPF recording applications, since its continuous mode feature can’t be operated at the designed 20 kHz sampling rate due to process variation. The future goal is to improve the channel sampling rate in continuous mode, so that the MINS IC can fully take advantage of the spatial resolution of the high density MEA, which is crucial for studying neural function at the network level. Preliminary work (Section 4.1.2) showed that the process variation error can be corrected by adjusting the IC bias current with off-chip real-time data acquisition and feedback circuits. However, the sampling rate is limited by the IC interface, which largely depends on the loading from the IC package and external components. Future iterations of the IC could include on-chip digitally programmable current trimming circuitry to avoid the timing overhead of communicating with off-chip circuits. Thus, it is feasible to scan the entire 256 channels in 50 μs (20 kHz sampling rate) and acquire the spatial information of neural activities recorded in continuous mode.

Finally, it became apparent from our measurements that the ability to adjust the gain of the recording channels would be of considerable value to prevent signals from saturation at the output. Such a gain adjustment could easily be integrated into the design of the MINS IC.

## 7. Future Work

For suggested future work, process variation correction circuitry can be integrated on-chip to improve the continuous mode scanning rate and reduce noise coupling to the bias current from the PCB. Also, the gain of the chip could be reduced to 40 dB (or preferably, made adjustable) for a better power/performance tradeoff, to reduce the power density, and to prevent the output signal from saturating for larger input signals. To further reduce the power density, the tail current should be turned off when a column is idle, and turned back on one cycle before the column is selected. Lead-free versions of C4s will be used to replace leaded C4s, and studies on the chronic effects of C4s on living animals will be carried out.
